# Transaminitis from Duloxetine: Case Report

**DOI:** 10.5811/cpcem.61865

**Published:** 2026-08-06

**Authors:** Gayle Galletta, Savant Mehta, Daniel Jancura, Daminica Ryan, Irene Li

**Affiliations:** * University of Massachusetts, Department of Emergency Medicine, Worcester, Massachusets; † University of Masachussetts, Department of Gastroenterology, Worcester, Massachusetts; ‡ University of Masachussetts, Worcester, Massachusetts

**Keywords:** duloxetine, transaminitis, liver injury, case report

## Abstract

**Introduction:**

Duloxetine, a serotonin-norepinephrine reuptake inhibitor, is commonly prescribed for depression, anxiety, and neuropathic pain. Although rare, duloxetine has been associated with hepatotoxicity.

**Case Report:**

We present the case of a 61-year-old male with multiple comorbidities who developed significant transaminitis two months after initiation of duloxetine therapy.

**Conclusion:**

Emergency physicians should be cognizant of duloxetine-induced liver injury, particularly in patients with increased risk factors who present with unexplained liver injury.

## INTRODUCTION

Duloxetine is a serotonin-norepinephrine reuptake inhibitor frequently used in the management of depression, generalized anxiety disorder, fibromyalgia, and diabetic peripheral neuropathy.^1^ Shortly after its Food and Drug administration (FDA) approval in 2004, post-marketing surveillance revealed reports of hepatotoxicity, ranging from mild liver enzyme elevation to severe hepatocellular injury with jaundice.^1^ Risk factors may include alcohol use, pre-existing liver disease, polypharmacy, and drug interactions.^1^ While there have been published case reports and series of duloxetine-induced liver injury,^2^–^7^ none have appeared in the emergency medicine literature. Here, we present a case of acute transaminitis associated with duloxetine, presenting to a community emergency department (ED).

## CASE REPORT

A 61-year-old male with a past medical history significant for type 1 diabetes, hypertension, coronary artery disease, remote bilateral below-the-knee amputations from gangrene while on vasopressors for diabetic ketoacidosis, remote cholecystectomy, hypothyroidism, celiac disease, anxiety, and depression presented to a community ED for elevated liver function tests. The patient had recently established care at a tertiary-care celiac disease clinic, where routine laboratory tests were performed days in advance of his first intake appointment, with results available on the day of presentation. He was referred to the ED because of the abnormal blood work.

His aspartate aminotransferase (AST) was 4,260 U/L (reference range, 8–48 U/L), and alanine aminotransferase (ALT) was 3,050 U/L (7–55 U/L). Alkaline phosphatase was 227 U/L (40–130 U/L). Total bilirubin was not tested. Other than his chronic diarrhea, attributed to celiac disease, and his chronic phantom- limb pain, the patient denied any physical complaints. He specifically denied abdominal pain, nausea, vomiting, or jaundice. He manages his diabetes with an insulin pump and takes acetaminophen 1,000 mg nightly. He had taken the once nightly acetaminophen for years for his phantom limb pain. He denied any additional dosages or taking other acetaminophen-containing products, and acetaminophen level resulted < 5 μg/mL (reference range, < 10 μg/mL). Other medications included aspirin, amlodipine, levothyroxine, methylphenidate, metoprolol, rosuvastatin, ticagrelor, and valsartan. Of note, the patient mentioned that he was started on duloxetine approximately two months earlier for depression. Duloxetine was initiated at 20 mg daily and increased monthly at 20 mg increments until the patient reached 60 mg daily. His last dose was the day before he presented to the ED. He rarely drank alcohol (< 1 drink per week) and denied ingestion of mushroom or other toxins. On arrival, he was afebrile and hemodynamically stable. His physical exam was unremarkable with no jaundice, scleral icterus, or abdominal tenderness.

The case was discussed with the hepatology service at our tertiary-care referral center with plans for transfer once a bed became available. We considered other etiologies of transaminitis including acetaminophen, ethanol, mushrooms, viral and autoimmune hepatitis, and gallbladder disease. Lab work was repeated in the ED five days after the initial labs, and showed a normal complete blood count; normal electrolytes; creatinine 1.07 (reference range, 0.6–1.3 mg/dL); blood sugar 157 (65–99 mg/dL); AST 132 U/L (10–40 U/L); ALT 619 U/L (10–40 U/L); total bilirubin 0.5 mg/dL (0.2–1.3 mg/dL); and international normalized ratio 1.0 (0.9–1.1). An ultrasound of the right upper quadrant showed hepatic steatosis but was otherwise normal. In addition, the hepatology service ordered tests to rule out viral and auto-immune hepatitis, which resulted days later. These included viral hepatitis panel, Epstein-Barr virus (EBV) antibodies, liver kidney microsomal antibody, and antinuclear antibody. These tests were negative, except for an elevated EBV immune globulin G, indicating prior infection.

Given the timing of initiation, dose titration, and absence of other etiologies, duloxetine-induced hepatotoxicity was suspected in the ED. Duloxetine was discontinued. Due to crowding at the tertiary-care referral hospital, a bed never became available for our patient. He was eventually discharged from the ED with outpatient follow-up.

## DISCUSSION

Duloxetine is metabolized primarily by cytochrome P450 family 1 subfamily A member 2 (CYP1A2) and CYP2D6.^1^ Hepatotoxicity is hypothesized to result from drug-drug interactions. Risk is higher in patients with alcohol use or underlying liver disease; therefore, duloxetine should be avoided in these populations.^8^

Case reports describe a wide latency period, with hepatoxicity occurring within three days of dose escalation or over five months after initiation.^2^–^4^ It can also occur in patients who have been maintained on the same regimen for years but are taking concomitant hepatotoxic medications.^5^ Most cases involve hepatocellular injury with marked transaminase elevation and, in some instances, jaundice or coagulopathy.^2^–^7^ Symptoms commonly reported include fatigue, anorexia, abdominal pain, or malaise, although our patient remained asymptomatic.^2^–^7^

The Roussel Uclaf Causality Assessment Method (RUCAM) is a scoring system used to determine the likelihood that a liver injury is due to a specific medication.^9^ It considers the time of drug initiation to liver injury, the response to discontinuing the suspected drug, risk factors such as age and alcohol use, concomitant use of hepatotoxic drugs or herbs, alternative causes of liver injury such as viral hepatitis, biliary obstruction, autoimmune liver disease, known information on a drug’s hepatotoxicity, and response to unintentional re-exposure (if applicable).^9^ Our patient scored eight on RUCAM, which indicates it was probable that duloxetine caused the liver injury (+2 for time of onset, +3 for decrease in ALT level > 50% within 8 days, +1 for > 55 years, −2 for concomitant acetaminophen use, +2 for exclusion of other causes of liver injury, +2 for known hepatotoxicity of duloxetine).

*CPC-EM Capsule*

What do we already know about this clinical entity?

*There are case reports of hepatotoxicity from duloxetine. The latency period is wide and the risk is greatest in patients with underlying liver disease or those taking other hepatotoxic substances.*

What makes this presentation of disease reportable?

*This is the first report of duloxetine hepatotoxicity in the emergency medicine literature.*

What is the major learning point?

*The Roussel Uclaf Causality Assessment Method (RUCAM) is a scoring system used to determine the likelihood that a liver injury is due to a specific medication. It takes into account the timing of drug initiation, concomitant use of hepatotoxic substances, and alternative causes of liver injury.*

How might this improve emergency medicine practice?

*Emergency medicine physicians should consider duloxetine as a possible etiology of transaminits.*

Our patient presented with liver function test abnormalities approximately two months after duloxetine initiation. His duloxetine regimen, 60 mg daily, is consistent with previously published cases of duloxetine-induced liver injury.^2^–^4^, ^6^,^7^ No alternative causes of liver injury were identified, raising concern for drug-induced liver injury. Current treatment focuses on the discontinuation of duloxetine and supportive care.^1^ In most published reports, liver enzymes improve within days to weeks after drug discontinuation.^2^,^3^ Severe cases may require hospitalization, but fulminant hepatic failure is rare.^4^,^6^

This case highlights the importance of monitoring liver function in patients on duloxetine, especially during the first months of therapy or after dose titration. Clinicians should maintain a high index of suspicion when evaluating unexplained transaminitis in patients taking duloxetine.

## CONCLUSION

We report a case of significant transaminitis occurring two months after initiation and titration of duloxetine in a patient with multiple comorbidities. Discontinuation of duloxetine resulted in laboratory improvement. This case reinforces the need for careful monitoring of liver function in patients receiving duloxetine, particularly those with additional risk factors. Emergency physicians need to be aware of duloxetine as a possible culprit in patients with transaminitis.

## References

[1] Eli Lilly (2010). Cymbalta (duloxetine).

[2] Kassam AS, Cunningham EA, Musco SE (2019). Dangers of rapid dosing: a case of dose-dependent drug-induced liver injury from duloxetine. Prim Care Companion CNS Disord.

[3] Kang SG, Park YM, Lee HJ (2011). Duloxetine-induced liver injury in patients with major depressive disorder. Psychiatry Investig.

[4] Vuppalanchi R, Hayashi PH, Chalasani N (2010). Duloxetine hepatotoxicity: a case-series from the drug-induced liver injury network. Aliment Pharmacol Ther.

[5] Malik B, Abdelazeem B, Revere T (2021). Duloxetine-induced liver injury: a case report. Cureus.

[6] Hanje AJ, Pell LJ, Votolato NA (2006). Case report: fulminant hepatic failure involving duloxetine hydrochloride. Clin Gastroenterol Hepatol.

[7] Park YM, Lee BH, Lee HJ (2010). Cholestatic jaundice induced by duloxetine in a patient with major depressive disorder. Psychiatry Investig.

[8] Duloxetine (2018). Liver Tox: Clinical and Research Information on Drug-Induced Liver Injury [Internet].

[9] (2019). LiverTox: Clinical and Research Information on Drug-Induced Liver Injury [Internet].

